# Gender and age concordance between patient and GP: an observational study on associations with referral behaviour

**DOI:** 10.3399/BJGPO.2022.0091

**Published:** 2022-11-02

**Authors:** Dorus Eggermont, Anton E Kunst, Karin Hek, Robert A Verheij

**Affiliations:** 1 Department of Public Health, Amsterdam UMC, University of Amsterdam, Amsterdam, The Netherlands; 2 Nivel, Netherlands Institute for Health Services Research, Utrecht, The Netherlands; 3 School of Social and Behavioral Sciences, Tranzo Tilburg University, Tilburg, The Netherlands

**Keywords:** gender concordance, age concordance, social concordance, decision making, referral decision making, referral and consultation, primary health care, medical specialist care, general practitioners, general practice

## Abstract

**Background:**

Appropriate referral from primary to secondary care is essential for maintaining a healthcare system that is accessible and cost-effective. Social concordance can affect the doctor–patient interaction and possibly also referral behaviour.

**Aim:**

To investigate the association of gender concordance and age concordance on referral rates in primary care in The Netherlands.

**Design & setting:**

Electronic health records data (*n* = 24 841) were used from 65 GPs in The Netherlands, containing referral information, which was combined with demographics of GPs and patients to investigate factors associated with referral likelihood.

**Method:**

Health records covered 16 different symptoms and diagnoses, categorised as ‘gender sensitive’, ‘age sensitive’, ‘both age and gender sensitive’, or ‘neutral’ based on Delphi consensus. Multi-level logistic regressions were performed to calculate the associations of gender and age concordance with referral status.

**Results:**

Overall, 16.8% of patients were referred to a medical specialist. The female–male dyad (GP–patient) was associated with a higher referral likelihood (odds ratio [OR] 1.14; 95% confidence interval [CI] = 1.02 to 1.27; *P* = 0.02) compared with the female–female dyad. Gender discordance was associated with a higher referral likelihood regarding consultations involving ‘gender-sensitive’ symptoms and diagnoses (OR 1.21; CI = 1.02 to 1.44; *P* = 0.03), and in duo and group practices (OR 1.08; 95% CI = 1.00 to 1.16; *P* = 0.05). Age concordance was not a significant predictor of referrals in the main model nor in subgroup analyses.

**Conclusion:**

Gender discordance was associated with a higher likelihood of referring. This study adds to the evidence that gender concordance affects decisions to refer, particularly with respect to symptoms and diagnoses that can be regarded as ‘gender sensitive’.

## How this fits in

Previous studies indicate that social concordance can affect doctor–patient interaction. However, research focusing on associations between social concordance and consultation outcomes, such as referral and/or prescription, are scarce. This study has shown that gender concordance can be associated with referral likelihood, underlining that medical decision making in the daily practice is subject to implicit social effects.

## Introduction

Appropriate referral from primary to secondary care is essential in maintaining a healthcare system that is accessible and cost-effective.^1^ Unnecessary referral is accompanied by inappropriate utilisation and distribution of medical resources,^2^ making it important to identify factors influencing decisions to refer patients to a medical specialist. In many countries (for example, The Netherlands, the UK) GPs have a gatekeeping role regarding access to secondary care.^3^

Referral decisions are about weighing medical, psychological, and social factors together with a patient. Moreover, this decision making often takes place in the context of patient pressure and time scarcity. Studies in the US and Norway showed that approximately half of the primary care physicians made unnecessary referrals in response to patient requests.^4,5^ A survey among Dutch GPs revealed that 71% of the physicians considered patient requests a barrier for preventing unnecessary care and 43% of GPs experienced too little time for shared decision making in order to limit unnecessary care.^6^ Referral decision making is thus complex, subject to time and patient pressure, and shows great variation between GPs.^7,8^

Social concordance is a construct describing shared identity based on demographic characteristics (for example, age, sex, ethnic group). Although the evidence is equivocal,^9,10^ social concordance can affect doctor–patient interaction.^9,11^ For example, ethnic concordant consultations are longer and show higher ratings of patient satisfaction, perceived quality, and shared decision making.^10,12–14^ Female concordance results in consultations containing more affective talk and less analytical talk,^15^ leading to female concordance being more patient-centred.^16–18^ Given that referral rates vary greatly between GPs and GPs experience patient pressure,^4–8^ social concordance may help GPs to find common ground more easily, thereby avoiding unnecessary referrals in response to patient request.

Social concordance may influence referral decision making because of different communication patterns.^16–18^ One possible reason for these communication differences is that social concordance makes it more likely that patient and doctor perceive each other as ‘in-group’ members, creating a platform for shared cognition^19^ and shared identity.^20,21^ Second, social concordance can also affect the communication by reducing professional uncertainty, as doctors treating patients who are more like themselves are less uncertain with respect to such person’s ailments and their treatment.^22^ Whether the influence of social concordance is necessarily mediated by a different communication style is debatable, since social concordance could also affect the doctor–patient interaction implicitly.^18,20,23,24^

This study investigated the effect of demographic GP and patient concordance on the likelihood of GP referral to secondary care. Overall, it was expected that social concordance would be associated with fewer referrals to secondary care. In addition, it was expected that concordance effects would be more present when symptoms or diagnoses can be regarded to be more prone to gender and age differences. Lastly, social concordance might become less relevant when the relationship between patient and doctor is stronger, as the authors consider to be the case in single-handed practices in comparison with duo or group practices.

## Method

### Data source

Electronic health records data were used from 2018 provided by practices participating in the Nivel Primary Care Database. These data were enriched with GP and practice characteristics of the participating practices (64 practices encompassing 158 GPs). The resulting dataset contained information on consultations, patients, GPs, and practices. Consultation data included the following: date; consultation type; referral information (did or did not refer to secondary care); and presenting symptom (coded with International Classification of Primary Care [ICPC] version 1). The ICPC classification system is used to record symptoms and/or diagnoses.^25^ Patient data included year of birth, sex, and presence of chronic disease (none versus one or more chronic diseases). GP and practice data included year of birth, sex, and type of practice (single-handed, duo, or group practice). The variable ‘gender concordance’ encompasses the following four groups: male–male dyads; female–female dyads; male–female dyads; and female–male dyads. The variable ‘age concordance’ encompasses the following three groups: age discordant dyads with patients >5 years younger than their GP; age discordant dyads with patients >5 years older than their GP; and age concordant dyads.

### Selection of consultations

Consultations were selected on the basis of the ICPC code that was recorded by the GP (Table 1). Symptoms and/or diagnoses presented in these contacts had to meet the following two criteria: (1) the ICPC code has relatively high referral rate (>5% of consultations with a referral to secondary care); and (2) the code has substantial absolute number of referrals (>100 referrals in the database). Consultations with the ICPC-code ‘Birth control IUD’ also met these criteria and were therefore also included. In order to differentiate between symptoms and diagnoses that could be more susceptible to effects of gender and/or age concordance, a Delphi consensus panel was organised with eight independent GPs to categorise the 16 ICPC codes as ‘gender sensitive’, ‘age sensitive’, ‘both age and gender sensitive’, or ‘neutral’ (Table 1). Supplementary Box 1 contains a more extensive description of the Delphi consensus panel.

**Table 1.** Symptoms and diagnoses of consultations included in the analyses

ICPC-code	Symptoms and diagnoses	Percentage of allselected consultations (*n*)	Referral rate(M = 16.8%) % (*n*)	Supposed higher relevance ofconcordance
**A04**	Tiredness or weakness	13.1% (2542)	8.7% (222)	Age and gender
**S99**	Other skin complaints	10.7% (2086)	15.5% (323)	None
**L99**	Other musculoskeletal complaints	9.9% (1935)	12.9% (250)	None
**D06**	Aspecific localised abdominal pain	9.9% (1926)	15.5% (299)	Gender
**S88**	Eczema or dermatitis	9.6% (1872)	9.8% (183)	Age
**L08**	Shoulder complaints	9.1% (1775)	12.3% (218)	None
**L15**	Knee complaints	8.5% (1664)	21.0% (349)	Age
**L86**	Lower back pain with radiation	6.5% (1274)	17.2% (219)	None
**W12**	Birth control or IUD	4.4% (858)	12.6% (108)	Non applicable
**S82**	Nevus or birthmark	4.3% (831)	22.5% (187)	None
**N01**	Headache	4.1% (791)	15.9% (126)	Age and gender
**L90**	Knee osteoarthritis	3.2% (630)	19.8% (125)	None
**F05**	Other visual complaints	2.4% (468)	60.9% (285)	Age
**K95**	Leg varicose	1.8% (357)	42.9% (153)	Age and gender
**H02**	Hearing complaints	1.4% (277)	41.2% (114)	None
**F04**	Floaters, flickers, or flashes	1.0% (191)	57.6% (110)	None
		100% (19 477)		

ICPC = International Classification of Primary Care. IUD = intrauterine device. M = mean.

Only consultations where the GP created the health record related to the visit were used. Health records not created but edited by the GP were not used because of the possible risk that the patient did not actually consult the corresponding GP (but instead, for example, a practice nurse). Furthermore, consultations with children (aged 0–17 years), e-consultations, and telephone consultations were excluded. Children were excluded because those consultations are frequently triadic instead of dyadic, complicating the interaction between child and GP.^26–28^

The initial dataset contained 73 897 consultations, 24 841 of which had an electronic health record created by a GP. After excluding 1989 consultations with children, 3114 phone consultations, and 261 e-consults, a total of 19 477 consultations (18 780 were in the practice and 697 were home visits) remained for analysis. These consultations were handled by 65 physicians in 25 practices.

### Statistics

To account for clustering of observations at the GP level, multi-level analysis was performed with patients nested in GP practices. GP practice was not taken into account as a separate level because there were too few GPs per practice. Generalised linear mixed models with binary logistic regression were performed. In the main model, referrals were predicted with gender concordance and age concordance as independent variables. Control variables were patient age, patient gender, GP age, GP gender, and presence of a chronic condition. Subgroup analyses involved separate models for consultations, which were considered age sensitive and gender sensitive. Also, separate models were calculated for consultations in single-handed practices and consultations in duo and/or group practices. In these subgroup analyses, gender concordant and discordant dyads were used as merged groups in order to maintain adequate group sizes. For sensitivity analysis, all models were also calculated with age concordance defined as a maximum age difference of 10 years between GP and patient. All analyses were performed using Stata (version 17.0).

### Privacy

Dutch law allows the use of electronic health records for research purposes under certain conditions. According to legislation, neither obtaining informed consent from patients nor approval by a medical ethics committee is obligatory for this type of observational studies containing no directly identifiable data.^29^

## Results

### Descriptive statistics

**Figure 1.**
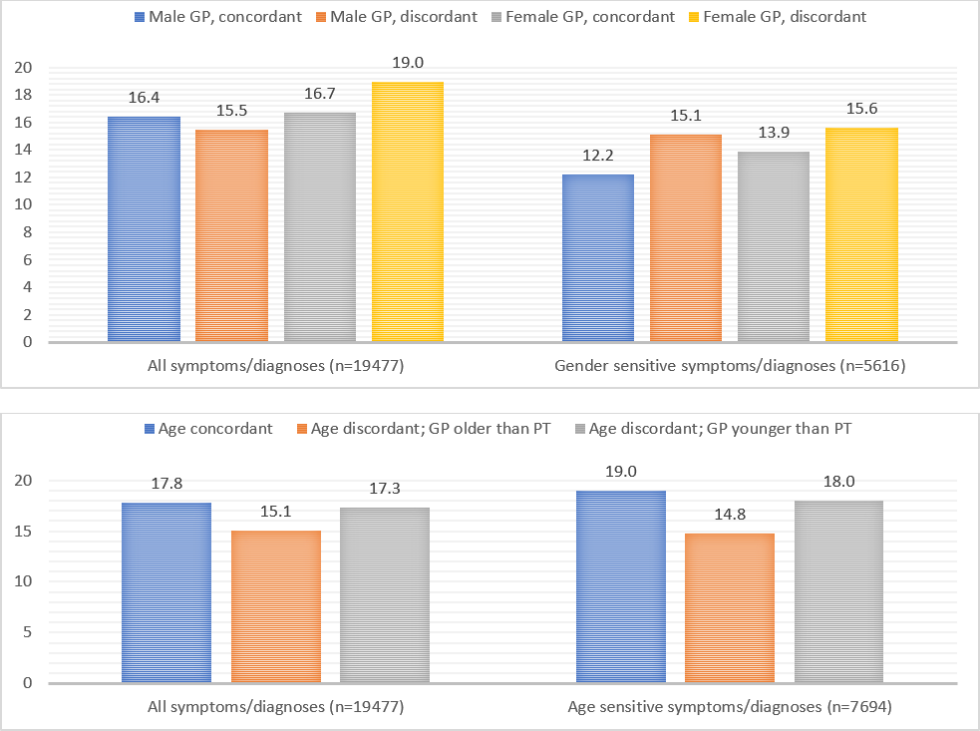
Referral percentage per gender and age dyad, stratified by type of ICPC-code. ICPC = International Classification of Primary Care. PT = patient.

The proportion of male GPs was 36.9%. Male GPs were on average older than their female colleagues (50 years versus 46 years). Seven GPs were in single-handed practices (10.8%), 24 GPs in duo practices (36.9%), and 34 GPs in group practices (52.3%). Of the 19 477 consultations, 36.6% involved a male patient. A slight majority of consultations were gender concordant (56.4%) and a minority of consultations were age concordant (18.0%). There were no differences in patient age and chronic conditions prevalence between the four gender dyads. Patient age and chronic conditions prevalence were different between all three age dyads (Table 2). In total, 16.8% of the patients were referred to secondary care. Figure 1 shows the referral percentage per gender and age dyad.

**Table 2.** Descriptives of gender dyads (GP and patient) and age dyads

	Male and male	Male and female	Female and female	Female and male	Age concordant	Age discordant:younger patient	Age discordant:older patient
Sample size	3492 (17.9%)	4908 (25.2%)	7499 (38.5%)	3578 (18.4%)	3498 (18.0%)	5098 (26.2%)	10 216 (52.5%)
Age GP, mean (SD)	50.5 (8.3)	50.8 (8.4)	45.8 (9.1)	45.7 (9.4)	49.1 (8.4)	52.0 (8.3)	45.5 (9.0)
Age patient, mean (SD)	56.4 (17.2)	54.8 (18.8)	52.6 (18.6)	56.5 (16.3)	49.4 (8.8)	33.5 (10.2)	67.3 (11.6)
Chronic conditions patient (%)	74.6	76.5	72.6	73.8	67.8	50.5	88.3
Referral rate (%)	16.4	15.5	16.7	19.0	17.8	15.1	17.3

### Main results

Gender discordance with a female GP was associated with more referrals to secondary care (OR 1.14; 95% CI = 1.02 to 1.27; *P* = 0.02) compared with the concordant dyads with a female GP (Table 3). For male GPs, referral likelihood did not differ between gender concordant and gender discordant dyads. Age concordance was not associated with referrals to secondary care. The age of the patient also predicted referral likelihood, with the youngest age group having the lowest referral likelihood and the middle-age groups having the highest referral likelihood. Chronic conditions and the age of the GP did not appear to be significant predictors in the model.

**Table 3.** Predictors for referral to secondary care

	All visits (*n* = 18 812)		
	Odds ratio	95% CI Exp(B)	Significance
Gender dyads			
Male GP – discordant	0.84	0.62 to 1.14	0.27
Male GP – concordant	0.88	0.64 to 1.21	0.42
Female GP – discordant	1.14	1.02 to 1.27	0.02
Female GP – concordant	1.00	–	–
Age concordance			
Discordant older GP	1.02	0.89 to 1.17	0.82
Discordant younger GP	0.99	0.86 to 1.13	0.83
Concordant*	1.00	–	–
Age GP			
30–39 years	1.00	–	–
40–49 years	1.03	0.73 to 1.45	0.88
>50 years	1.18	0.92 to 1.52	0.19
Age patient			
18–29 years	1.00	–	–
30–49 years	1.40	1.20 to 1.64	0.00
50–69 years	1.60	1.31 to 1.95	0.00
≥70 years	1.31	1.03 to 1.66	0.03
No chronic condition	0.91	0.81–1.02	.09

*Age concordance was defined as a maximum age difference of 5 years between GP and patient.

In the model with gender-sensitive symptoms and diagnoses (Table 4), gender concordance was a significant predictor for referral, leading to a higher referral likelihood when there is gender discordance (OR 1.21; 95% CI = 1.02 to 1.44; *P* = 0.03). In the model with age-sensitive symptoms and diagnoses, only patient age was a significant predictor for referral (in a similar pattern as observed in the whole study population). Age concordance and gender concordance did not predict referral likelihood in this subgroup.

**Table 4.** Subgroup analyses with age and gender-sensitive ICPC codes

	ICPC-code 'age' (*n* = 7451)			ICPC-code 'gender' (*n* = 5423)		
	Exp(B)	95% CI Exp(B)	Significance	Exp(B)	95% CI Exp(B)	Significance
Gender concordance						
Discordant dyads	1.09	0.98 to 1.20	0.10	1.21	1.02 to 1.44	0.03
Concordant dyads	1.00	–	–	1.00	–	–
Age concordance						
Discordant older GP	0.90	0.74 to 1.10	0.31	0.90	0.69 to 1.17	0.42
Discordant younger GP	0.94	0.79 to 1.12	0.49	0.86	0.68 to 1.10	0.24
Concordant*	1.00	–	–	1.00	–	–
Gender GP						
Male GP	0.86	0.61 to 1.23	0.42	0.87	0.61 to 1.24	0.44
Female patient	1.00	–	–	1.00	–	–
Gender patient						
Male patient	1.03	0.92 to 1.14	0.64	0.92	0.78 to 1.09	0.35
Female patient	1.00	–	–	1.00	–	–

The covariates Age GP, Age patient, and Chronic condition are not shown in this table, but were part of the calculated model. ICPC = International Classification of Primary Care.

* Age concordance was defined as a maximum age difference of 5 years between GP and patient.

Gender concordance and age concordance are not associated with referral likelihood in single-handed practices (Table 5). In duo and group practices, gender discordance is associated with a higher referral likelihood (OR 1.08; 95% CI = 1.00 to 1.16; *P* = 0.05). Age concordance did not predict referrals in single-handed or duo and group practices. In both single-handed practices and duo or group practices, male patients had a higher likelihood of being referred to secondary care, which was statistically significant in the latter group (OR 1.08; 95% CI = 1.00 to 1.17; *P* = 0.04).

**Table 5.** Stratification single-handed versus duo and group practice

	Consultations in solo practice (*n* = 2602)			Consultations in duo and group practice (*n* = 16 210)		
	Exp(B)	95% CI Exp(B)	Significance	Exp(B)	95% CI Exp(B)	Significance
Gender concordance						
Discordant dyads	0.88	0.72 to 1.08	0.22	1.08	1.00 to 1.16	0.05
Concordant dyads	1.00	–	–	1.00	–	–
Age concordance						
Discordant older GP	1.09	0.80 to 1.48	0.59	1.01	0.87 to 1.17	0.91
Discordant younger GP	1.01	0.90 to 1.14	0.85	0.98	0.83 to 1.16	0.83
Concordant*	1.00	–	–	1.00	–	–
Gender GP						
Male GP	0.54	0.24 to 1.26	0.15	0.80	0.58 to 1.11	0.19
Female patient	1.00	–	–	1.00	–	–
Gender patient						
Male patient	1.12	0.92 to 1.36	0.27	1.08	1.00 to 1.17	0.04
Female patient	1.00	–	–	1.00	–	–

The covariates Age GP, Age patient, and Chronic condition are not shown in this table, but were part of the calculated model.

* Age concordance was defined as a maximum age difference of 5 years between GP and patient.

## Discussion

### Summary

The objective of this study was to explore whether the likelihood of referral is associated with gender concordance and age concordance. It was found that discordance with a female GP (female–male dyad) is associated with more referrals to secondary care compared with concordance with a female GP (female–female dyad). In consultations containing gender-sensitive symptoms and diagnoses and in consultations within duo and group practices, gender discordance was associated with a higher referral likelihood. Age concordance was not associated with referrals to secondary care in all models.

### Strengths and limitations

Routinely recorded electronic health records were used, so the study benefited from having a large-scale representative sample. The data can be assumed to reflect daily practice without doctors and patients being aware of being observed. Because such data are not primarily intended for research, consistency of data quality is not necessarily guaranteed.^30^ It is, however, unlikely that data quality issues are systematically clustered within certain dyads, thus affecting the results.

Ideally, the authors would have liked to have incorporated aspects of practice location in the analyses (city, rural, and so on) to assess possible confounding. Moreover, three out of four patients had a chronic condition registered in their record, suggesting that the label chronic condition also includes mild conditions. It is unlikely that this would have affected the findings concerning the associations between gender concordance, age concordance, and referral likelihood.

Lastly, it should be noted that in this study referrals are being treated as a homogenous outcome. However, in actual practice referrals are highly heterogeneous. Depending on the type of symptom, the referral purpose (for example, diagnostic or therapeutic), level of urgency and so on, one referral is not necessarily interchangeable with another.

### Comparison with existing literature

It was expected that social concordance would influence referral decision making. By applying different communication patterns, possibly as a result of both more shared identity and less professional uncertainty, gender concordance could reduce referral likelihood. Former studies show varying results, but overall, the female–female dyad is found to have associations with several consultation outcomes^31–33^ (for example, treatment of diabetes, antibiotic prescription) and has been identified as having distinct communication patterns^15^ and being more patient-centred^16–18^ compared with other gender dyads. These observations are in line with the finding that the association between gender concordance and referral likelihood is most clear in dyads with a female GP. The present study's finding that female gender concordance can decrease referral likelihood corresponds with a former study showing that alignment in attitudes is associated with fewer referrals.^34^ The association of gender concordance with referral likelihood was stronger for symptoms and diagnoses that were regarded as more gender sensitive, adding to the plausibility of gender concordance influencing referral likelihood.

The association between gender concordance and referral likelihood was only apparent in duo and group GP practices. A possible explanation is that in single-handed practices patients and GPs have more trusted relationships in which concordance is of less importance. Also, having contact with the same caregiver for a longer period of time decreases medical specialist referral likelihood.^35^ Arguably, gender concordance is more likely to have an effect when patients and GPs are relatively unfamiliar with each other. When relationships become more established and there is more mutual trust, such factors could become less relevant.

Age concordance was not associated with referral likelihood. Research on the association between age concordance and healthcare consultation outcomes is scarce. In an observational study, age concordance was a component of the social concordance construct (also entailing race, gender, and education concordance). This study showed that social concordance was associated with higher satisfaction of care, but associations between the individual components and the defined outcome measures were not reported.^11^ Possibly, approximation in age is less substantially related to perceived similarity and different communication patterns as gender concordance.

Gender discordance was associated with a higher referral likelihood. However, it is difficult to draw conclusions about the appropriateness of a referral. Studies show that 23% to 37% of referrals to secondary care were considered unnecessary by the specialist.^36–38^ In contrast, 95% of patients rate referrals as necessary,^39^ illustrating that the judgement about the appropriateness of a referral is highly dependent on the person making the judgement. It was assumed that gender concordance service mutual understanding and common ground, thereby reducing unnecessary referrals in response to patient requests. This helps to avoid unnecessary costs (for patient and society) and unjustified allocation of scarce resources, making the findings clearly relevant.

### Implications for research and practice

Gender discordance was associated with a higher likelihood of referring, especially with health symptoms and diagnoses, which were regarded as gender sensitive, in duo and group practices and only when the GP was female. Possibly, higher referral rates in gender discordant dyads can be avoided by applying more patient-centred communication. Also, attempting to assign patients with gender-sensitive complaints to a GP with the same gender could potentially lower referral likelihood. Observational and/or qualitative research is needed to further examine what actually happens in the consultation room that could explain the findings and better understand the underlying mechanism behind effects of demographic concordance.

## Data availability

Data is available on request and under conditions as layed down in the governance code of Nivel-PCD.

## Data

The dataset relied on in this article is available from the corresponding author on reasonable request.
